# Sex Difference in Trigeminal Neuropathic Pain Response to Exercise: Role of Oxidative Stress

**DOI:** 10.1155/2020/3939757

**Published:** 2020-06-28

**Authors:** Zahra Rostami, Sahar Ghasemi, Hamed Farzadmanesh, Manouchehr Safari, Ali Ghanbari

**Affiliations:** ^1^Student Research Committee, Semnan University of Medical Sciences, Semnan, Iran; ^2^Department of Anatomical Sciences, Faculty of Medicine, Semnan University of Medical Sciences, Semnan, Iran; ^3^Research Center of Physiology, Semnan University of Medical Sciences, Semnan, Iran

## Abstract

**Aim:**

Orofacial chronic neuropathic pain commonly occurs following trigeminal nerve injuries. We investigated whether swimming exercise can reduce trigeminal neuropathic pain through improving antioxidant capacity.

**Materials and Methods:**

Twenty-eight Wistar rats of either sex and 180–220 grams were divided into 4 groups as sham, neuropathy, neuropathy + single bout exercise, and neuropathy + 2 weeks of exercise. Trigeminal neuropathy was carried out through chronic constriction injury (CCI) of infraorbital nerve. Protocols of exercise were included a single bout session (45 minutes) and a 2-week (45 minutes/day/6 days a week) swimming exercise. Mechanical allodynia was detected using Von Frey filaments. The activity of the serum antioxidant enzymes glutathione peroxidase and superoxides dismutase was assayed using ELISA kits.

**Results:**

We found that CCI significantly reduced facial pain threshold in both sexes (*P* < 0.05). Both swimming exercise protocols significantly reduced mechanical allodynia in female rats compared to the sham group; however, only 2 weeks of exercise were significantly effective in male rats. The activity of antioxidant enzyme glutathione peroxidase significantly (*P* < 0.05) decreased following CCI in female rats against that in the sham group and 2-week exercise significantly (*P* < 0.05) increased it toward the control level. The levels of glutathione peroxidase in male rats and superoxidase dismutase in both sexes were not significantly different compared to their sham groups.

**Conclusion:**

Swimming exercise alleviates trigeminal neuropathic pain in both sexes. Oxidative stress as a possible mechanism was involved in the effect of exercise on female rat trigeminal neuropathy.

## 1. Introduction

Neuropathic pain is a treatment-resistant outcome of injury to the central or peripheral nervous system, which has several behavioral signs, including mechanical allodynia and hyperalgesia, thermal hyperalgesia, and spontaneous pain [1].

Sex difference is a known factor in the pathology of chronic pain [2], which affects behavioral response to painful and nonpainful stimuli. The prevalence of the neuropathic pain is less common in male than in female subjects (3 in women against 2 in men) [3]. Diabetic males report a lower frequency and intensity of pain despite more severe polyneuropathy than females [4]. On the other hand, in animal study, lysophosphatidic acid- (LPA-) induced neuropathic pain is more pronounced in male rats than in female ones [5].

Oxidative stress as a mechanism in different health problems [6] has a possible role in the neuropathic pain.

Oxidative stress is a physiological pathway, and its imbalance (increased production of oxidant agents greater than the ability of endogenous antioxidant capacity to scavenging them) plays an important role in the pathogenesis of neural injuries [7]. Mammalian peripheral nerves are vulnerable to oxidative stress reactions due to high content of phospholipids, mitochondria, and weak cellular antioxidant capability [8]. The main biological oxidants are reactive oxygen species (ROS) which can promote reactions that lead to molecular instability including lipid peroxidation, DNA damage, and apoptosis [9]. ROS are capable of activating intracellular signaling pathways resulting in nuclear factor-*ĸ*B (NF-*κ*B) transcription which in turn leads to transcription and production of proinflammatory cytokines such as TNF-a that exacerbate the conditions [10].

Considering the beneficial effects of alpha-lipoic acid and vitamin C against oxaliplatin-induced hyperalgesia [11], it is concluded that oxidative stress plays a role in the neuropathic pain. Increased free radicals during diabetes disease are one of the possible mechanisms of damage to nerve fibers and abnormal nerve function [12]. Kallen Born and his colleagues in 2013 showed the role of ROS in neuropathic pain [13]. Apart from ROS production, nerve injury increases neurotrophic growth factors which lead to neuropathic pain through various mechanisms including upregulation of pain-related genes, amplification of glutamatergic synaptic transmission, glial cells activation in DRG, and increasing intracellular signaling molecules such as PLC*ʏ*-1 (phospholipase C gamma 1), ERK1,2 (extracellular signal-regulated kinases), and CREB (cAMP response element-binding protein) [14–18].

Despite the great progress in medicine, treatment of neuropathic pain is one of the major challenges for medical practitioners yet. Different involved mechanisms in neuropathic pain and altering over time [19] are a possible reason for failure to find a definitive permanent treatment. On the other hand, the extent of involved mechanisms leads to complications in pain management.

Common pharmacological methods have a temporary effect on pain [20, 21] and, moreover, the adverse effects of some of drugs have limited their use [22, 23]. Regarding the absence of a definitive treatment for pain, study on the nonpharmacological methods such as dietary, phytomedicine, and exercise to improve neuropathic pain is of great importance.

Exercise, particularly aerobic types, has improving effect on the neuropathic pain [24]. Scheduled physical activity reduces diabetic neuropathic pain [25]. Kuphal and his colleagues found swimming exercise reduces rat's pain response to formalin test [26]. Martinz et al. showed high-intensity swimming exercise has an analgesic effect in mice involved with complex regional pain syndrome-I (CRPS-I) [27]. It has been reported that voluntary exercise (wheel running) has antinociceptive effect against muscle noxious insult in mice [28]. Numerous mechanisms have been proposed on the effect of exercise on neuropathic pain such as decreased expression of glial cells markers, proinflammatory cytokines suppression, and BDNF release and oxidative stress inhibition in dorsal root ganglion and dorsal horn neurons [29–33]. Regular exercise induces the endogenous antioxidant system, which may protect the body from consequences of injuries caused by oxidative stress [34].

Considering the above, the aim of the present study was to evaluate exercise-induced hypoalgesia in both sexes following infraorbital nerve (IoN) chronic constriction injury (CCI) and whether oxidative stress plays a role.

## 2. Materials and Methods

### 2.1. Animals

In the present study, Wistar rats of either sex (28 male and 28 female rats), weighing 180–220 g, which were housed at a place with controlled temperature (20–24°c) and 12 h light-12 h dark cycles were used. Food and water were freely available to animals. Both sexes were divided into four groups as sham, neuropathy, neuropathy + single bout exercise, and neuropathy + 2 weeks of exercise. Sample size in behavioral experiments was 7 in each group, and in biochemical tests, it was 4 in each group. Present research was approved by the Ethics Committee of the Faculty of Medicine, Semnan University of Medical Sciences (Number IR.SEMUMS.AC.REC.1397.34). All of the experimental methods were conducted according to National Institutes of Health guidelines for working with laboratory animals. All experiments were carried out in a quiet room between 14 PM and 17 PM to avoid diurnal variations. The authors attest that all efforts were made to minimize the number of animals used and their suffering.

Experiments were performed according to the following timeline.

### 2.2. Surgery Procedure to Induce Neuropathic Pain

Chronic constriction injury of IoN was made by the method described by Ding [35] with little modification (IoN was legated using catgut chromic 6.0 in place of 4.0). After anesthetizing the rats using ketamine (80 mg/kg) and xylazine (10 mg/kg), a 0.5 cm incision was made on the face at the place of distal portion of left IoN in a line connecting inner corner of the eye to third row of whiskers. After exposing the IoN and isolating from surrounding tissues, two loose ligations were made around the nerve at 1-millimeter interval. Then, the skin was stitched using silk sutures 4.0. All animals were housed in isolated cages for one day to begin eating and drinking. Animals in the sham group received all of the procedure except nerve ligation.

### 2.3. Protocol of Exercise

Swim exercise was performed through the method described by Badreldin [36]. Animals swam 45 minutes daily in a plexiglass pool (depth: 65 cm, diameter: 50 cm) which contained 35 ± 1°c water. Two exercise programs were performed: the first one was a single session (45 minutes) swimming and the second one was a 2-week (45 minutes/daily/6 days a week) swimming exercise. In order to get familiar with the swimming exercise condition, the week before the main exercise program, rats were swimming 10 minutes at the first day and then 10 minutes added to swimming time daily until it reaches 45 minutes within one week. Two of the animals (both of them are female rats, one in single bout exercise group and another in 2-week exercise group) that were unable to continue exercise were excluded from study.

### 2.4. Evaluation of Pain-Like Behavior

Mechanical allodynia was evaluated at the whisker pad on the injured side using Von Frey hairs (Stoelting, Wood Dale, IL, and USA) [37]. These hairs are calibrated filaments and according to their diameter make a definite force (gram) on the surface which they were applied on. The hairs were used in ascending manner and were started with smallest force. If that filament did not stimulate a painful reaction, a stronger one would be used. Each filament was tested 5 times. Withdrawal reaction of face or attacking to filament in three times per five applied stimuli was considered as a response. All experiments were performed between 14 PM and 17 PM to avoid diurnal variations.

### 2.5. Biochemical Tests

Biochemical tests included glutathione peroxidase and superoxide dismutase assays in the serum.

### 2.6. Serum Preparation

After anesthesia, using a 5-milliliter syringe, blood sampling was carried out through the heart and centrifuged at 3000 rpm for 15 minutes. Serum was removed and kept in −80°C until biochemical experiments.

### 2.7. Glutathione Peroxidase and Superoxide Dismutase Assay

Glutathione peroxidase (GPX) activity and superoxide dismutase (SOD) activity were measured according to manufacturer's instruction of related kits ((ZellBio GmbH, Germany, Cat. No : ZB-GPX-A96) (ZellBio GmbH, Germany, CAT No. ZB-SOD-96A)) on the basis of colorimetric assay.

### 2.8. Data Analysis

Behavioral results were analyzed using one-way ANOVA and post hoc Tukey's test. Kruskal–Wallis test and Dunn's post hoc test were used to analyze the biochemical data. All of the data were expressed as mean ± SEM of measured parameter. *P* < 0.05 was considered as significant. The GraphPad prism 5.0 statistical software (GraphPad, San Diego, CA, USA) was used to analyze the data.

## 3. Results

Results of the present study revealed that swimming exercise attenuates facial neuropathic pain induced by CCI of infraorbital nerve through antioxidative stress effect.

### 3.1. Behavioral Results

**Figure 1:**
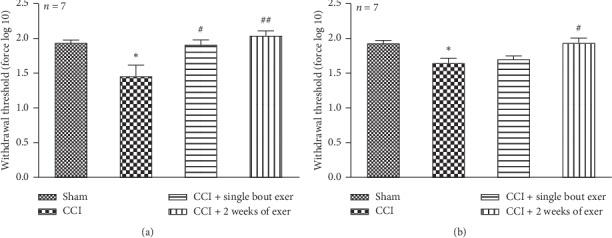
Effect of swimming exercise on the neuropathic pain induced by chronic constriction injury of infraorbital nerve in female (a) and male (b) rats. Neuropathy significantly reduced pain threshold response compared to the sham group in both sexes. Single bout exercise significantly increased pain threshold against that in the neuropathy group in female rats (a) only. Two weeks of exercise following neuropathy significantly increased pain threshold response against that in the neuropathy group in both sexes. All of the data are expressed as mean ± SEM (*n* = 6 per group). “Asterisks” is used to compare the CCI group with respect to the sham group and “#” sign is used to compare the exercise group against the CCI group. ^*∗*^*P* < 0.05, ^#^*P* < 0.05, and ^##^*P* < 0.01.

Our results showed that swimming exercise increases face withdrawal threshold in IoN CCI female and male rats (Figure 1). Chronic constriction injury of female IoN significantly decreased face withdrawal threshold compared to the sham group (1.45 ± 0.16 in the CCI group against that 1.93 ± 0.04 in the sham group) (Figure 1(a)). Face withdrawal threshold in female rats significantly increased following both single bout exercise (mean ± SEM = 1.9 ± 0.069, F _(1,15)_, *P*=0.04) and 2 weeks of exercise [mean ± SEM = 2.04 ± 0.07, F _(1,16)_, *P*=0.004]; however, observed hypoalgesic effects in male rats (Figure 1(b)) were smaller than in female rats. In single bout exercise [F _(1,14)_, *P*=0.1] and in 2-week exercise [F _(1,15)_, *P*=0.011]. Chronic constriction injury of male IoN significantly decreased face withdrawal threshold compared to the sham group (1.65 ± 0.07 in the CCI group against that 1.93 ± 0.04 in the sham group) (Figure 1(b)).

### 3.2. Biochemical Results

**Figure 2:**
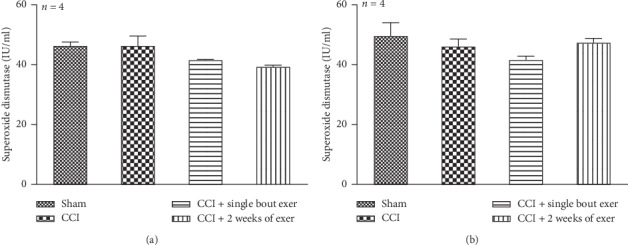
Effect of swimming exercise on the serum level of superoxidase dismutase enzyme in the neuropathic female rats (a) and neuropathic male rats (b). Serum superoxidase dismutase level did not significantly change against its control level in female rats (a) and male rats (b) (*n* = 4 per group). All of the data are expressed as mean ± SEM.

We measured antioxidant enzymes, superoxide dismutase (SOD), and glutathione peroxidase (GPx) to determine whether oxidative stress has a role in the IoN CCI-induced neuropathic pain. We found that serum SOD activity levels were not significantly different between the CCI group and the sham control group in both sexes. Swimming exercise did not change SOD activity compared to the CCI group neither in female (Figure 2(a)) nor in male (Figure 2(b)) rats.

**Figure 3:**
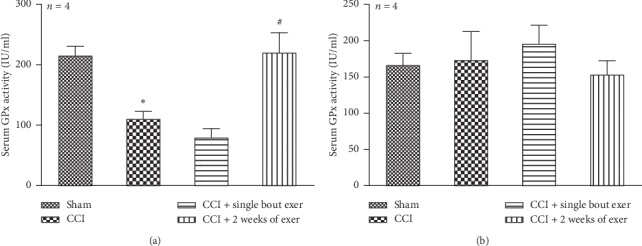
Effect of swimming exercise on the serum glutathione peroxidase (GPx) level with respect to the control group. Neuropathy of infraorbital nerve in female rats significantly reduced the GPx level in serum compared to the sham group and 2 weeks of swimming exercise reversed it toward the sham control group (a). Glutathione peroxidase levels were not significantly different between sham, neuropathy, and exercise groups in male rats (b). All of the data are expressed as mean ± SEM (*n* = 4 per group). “Asterisk” is used to compare the CCI group in comparison with the sham group, and “#” sign is used to compare the exercise group against the CCI group. ^*∗*^*P* < 0.05 and ^#^*P* < 0.05.

Results of GPx activity in our experiments showed that chronic constriction injury of IoN in female rats significantly (*P* < 0.05) reduced GPx activity compared to the sham control group and swimming exercise reversed it toward control level (Figure 3(a)). Kruskal–Wallis test showed two weeks of swimming exercise but no single bout swimming exercise (222 ± 33 in the two-week exercise group versus 110.5 ± 12.3 in the CCI group, *P*= 0.013) significantly increased GPx activity compared to the CCI group (Figure 3(a)). However, GPx activity in male rats showed no significant difference between groups (Figure 3(b)).

## 4. Discussion

Trigeminal neuropathic pain is a debilitating problem in the orofacial region, which is caused by several reasons such as traumatic events, dental and facial surgeries, and viruses' attacks [38–40]. Despite the current medical treatments and even surgical approaches applied for trigeminal neuropathy, orofacial neuropathic pain is a challenge for medical practitioners yet.

Regular physical exercise has been revealed to reduce the severity of neuropathic pain. In the present study, the effect of swimming exercise on the facial neuropathic pain induced by chronic constriction injury of IoN was evaluated on rats of both sexes.

Our results showed that CCI on infraorbital nerve led to facial neuropathic pain. This result is consistent with the results of other researchers, who reported the neuropathic pain at the IoN territory following CCI of infraorbital nerve [35, 41]. In our experiments, swimming exercise improved infraorbital nerve CCI-induced facial neuropathic pain. We evaluated two exercise regimens, single bout swimming and 2-week (six days per week) swimming exercise. We found that single bout swimming exercise alleviates neuropathic pain in female rats but not in male rats; meanwhile, 2-week exercise was effective in both sexes. Our result of female single bout exercise is in agreement with other studies, which revealed hypoalgesic effect following single session exercise [42, 43]; however, other studies showed that single bout exercise has hypoalgesic effect in both sexes [44]. There are several reports about involved mechanisms in the exercise-induced hypoalgesia among which release of endogenous opioids has a great attention [45, 46]. It has been reported that beta-endorphins level in the blood increases following exercise [45]. Moreover, opioid antagonist (naloxone) administration before mild swimming exercise prevents exercise-induced hypoalgesia [47]. In contrast, some studies showed that opioidergic system does not have a role in exercise-induced hypoalgesia [48]. On the other hand, the effect of the endocannabinoid system is suggested as another related mechanism in hypoalgesic effect of acute exercise [49, 50].

We showed that the pain threshold difference between the exercise group and the IoN CCI group was significant in female rats. Male rats did not show hypoalgesia following single bout exercise and their hypoalgesic response to two-week exercise program was not as significant as female rats (*P* < 0.05 in male rats versus *P* < 0.01 in female rats). Our result is in agreement with human and animal studies about sex difference in pain behavior in response to injury and pharmacologic treatment [37, 51]. Several studies showed more hypoalgesia following exercise in female than male human subjects which their baseline pain threshold was not significantly different [52–54]. Moreover, it has been shown that a multidisciplinary pain treatment program improved pain induced disability in women more than men [55]. In the research of Sternberg and his colleagues, they observed a significant increase in pain threshold following treadmill exercise in female subjects but not in males [56]. There are several reports about release and antinociceptive effects of endogenous opioids following exercise [57, 58]. On the other hand, Chakrabarti and his colleagues showed that expression of opioid receptors (*μ*- and *κ*-opioid receptors) is more in female than male rats (a biological difference) [59]. A meta-analysis study revealed more analgesia following morphine consumption in female patients than male patients [60]. Considering these, it is possible that in our experiment, higher expressions of opioid receptors in female rats along with opioid release following exercise led to more analgesia in female rats than male rats. Dominiguez and his colleagues reported that female rats are more sensitive than male rats to IoN injured pain [36]. Although we found pain threshold to injury was lower in female rats, however, there was no significant difference between both sexes. This result is consistent with other results which showed no significant difference between pain response in males and females [53, 54].

There are several reports that showed that mild-to-moderate intensity exercise has more positive effects on females than males [61–63]. Generally, men have better emotional feelings, lower depression, anxiety, and somatotopic complications following high intensity exercise; meanwhile, women gain more reduction in physical symptoms of somatic problems following light or medium intensity exercise [64]. Considering this, it seems that female responds to medium intensity of physical activity better than male. Regarding higher basal level of activity in male than female (gender difference) [64], it seems that at the same medium level of intensity, female has clearer response to physical activity than male. Therefore, gender difference is an issue that affects pain response of either sex to stimulus which should be considered.

In the present study, we examined antioxidative role of exercise through measuring GPx and SOD activity level in the serum. It has been suggested that hypoalgesic effect of exercise is related to its global antioxidative and anti-inflammatory effect [65, 66]. Hassler and his colleagues pointed out the role of antioxidants in relieving neuropathic pain [67]. They reported that intrathecal injection of apocynin, an inhibitor of NADPH oxidase, alleviates mechanical allodynia in spinal cord injured rats through reduction of ROS production.

In the present study, we showed that 2-week exercise significantly increased GPx antioxidant enzyme activity in female rat's serum. However, exercise did not change GPx activity in CCI male rat's serum compared to the control group. Our result is consistent with report of Yamamoto and his colleagues which showed liver GSH and GPx activity in voluntary exercised female rats was significantly higher than in male rats [68]. Further, Balci and colleagues reported that high-intensity exercise increased gastrocnemius GSH level more in female rats than in male rats [69]. On the other hand, it has been reported that GPx activity in the female rat liver mitochondria is more than those in male rat's liver [70]. Also it has been shown that production of H_2_O_2_ in the liver and brain mitochondria of female rats is more than in male rats [71]. Moreover, Lim and colleagues showed that female mice myocardium is more resistant against ischemia/reperfusion injury than those in male mice due to greater capacity of their antioxidant system [72].

In our experiments, SOD level in the CCI group was similar to the control group and swimming exercise does not alter its level compared to the CCI group in both sexes. In agreement with this, research of Balci and his colleagues showed no significant difference in SOD level of gastrocnemius and heart tissues of female and male rats following endurance exercise [69]. There are several reports that showed SOD level does not change following exercise [73, 74]. Wiecek and colleagues in 2018 reported that blood SOD level showed similar changes following anaerobic exercise in women and men [75]. In contrast to our result, it has been reported that SOD level was significantly higher in female than in male before and after exercise [76]. Generally, there are some controversial reports about gender differences of SOD. Some people argued that based on the evaluated tissues, SOD level may have gender difference. As Chen reported, SOD activity in female mice brain and lung is greater than those in male mice brain and lung, but its level in the heart and kidney is identical in both sexes [77]. However, discrepancy in literatures may be due to various methods to detect SOD activity, different types of applied exercise and program, and difference in the types of tissues and sexes studied.

According to various studies which showed higher concentration and/or higher activity of antioxidant enzymes in females versus males [71, 78, 79], it seems that antioxidant systems of females are more ready to involve oxidative stress issues than males.

Unfortunately, in the present study, due to financial limitation, we could not measure ROS level/activity in the experimented animals. If we could detect ROS levels, we would enable presenting a more precise interpretation.

There are several reports toward involved mechanisms in exercise-induced analgesia exclusive than oxidative stress. Exercise through reducing excitatory glutamatergic transmission leads to antinociception [80]. Further, exercise reverses increased BDNF and NGF levels in DRG following nerve injury. Moreover, it has been reported that exercise normalizes PLCʏ-1 phosphorylation level which is necessary for stimulation of glutamate release by BDNF through TRK/PLC_Y_-1 pathway [81, 82]. Considering the multiplicity of mechanisms involved in exercise-induced-analgesia, some of which were mentioned, it would be valuable if we could evaluate neurotrophic factors and/or intracellular signaling molecules. Since, in our country, we have severe financial problems, we were unable to evaluate further parameters other than antioxidative enzymes, which is one of the major limitations of present study.

However, gender and sex are an important matter that should be noticed in pain investigations. Sex differences have been considered in many fields of biological research. Despite the numerous studies emphasizing sex difference in pain behavior response [83], there are many investigations that reported similar pain sensitivity in both sexes [84].

Pain is a complicated condition which is impressed by factors such as sex (in points of view such as biological, psychological, and social situations) and different pathophysiological mechanisms (between cephalic and extracephalic nerves), and therefore the results of pain study should be carefully interpreted.

## 5. Conclusion

Swimming exercise with medium intensity reduces CCI-induced trigeminal neuropathic pain more clearly in female rats than male rats, and hypoalgesia in female rats possibly occurs through antioxidative property of exercise.

## Data Availability

The original data used to support the findings of this study are available from the corresponding author upon request.
